# Collaborative interventions with law enforcement in the management of childhood trauma

**DOI:** 10.1192/j.eurpsy.2025.1110

**Published:** 2025-08-26

**Authors:** T. Bollain Muñoz, I. Oliveira Amat, R. Crespo Rubio, T. Gomez Escribano, J. Gomez Puebla, J. M. Martinez Avila

**Affiliations:** 1 Psychiatry, HGUGM, Madrid, Spain

## Abstract

**Introduction:**

Studies indicate that approximately 50% to 70% of children will experience at least one adverse childhood event, such as abuse, neglect, household dysfunction, community violence, or natural disasters.

Yet, accutely traumatized children are rarely seen in outpatient clinics until months or even years later, often presenting with chronic symptoms or maladaptive behaviors. Exposure to adverse childhood experiences (ACEs) has been linked to a wide range of psychiatric disorders, including mood disorders, personality disorders, and substance abuse. Beyond mental health, ACEs have been correlated with higher rates of chronic medical conditions such as heart disease, metabolic disorders, and autoimmune diseases, as well as a reduced life expectancy. Furthermore, children who are abused are more likely to perpetuate abuse in adulthood, leading to a cycle of violence that spans generations. Given its widespread and long-lasting effects, childhood trauma is indeed a public health pandemic.

**Objectives:**

Enhancing our response to and prevention ACEs requires a multisectoral approach.

**Methods:**

One key approach is a collaborative response with law enforcement. Police officers have unique access to children at risk, but they often lack the specialized training to respond effectively. With appropriate training, law enforcement can serve as a critical bridge to mental health assessments for children and their families.The Child Development-Community Policing Program exemplifies such collaboration. This initiative, a partnership between the New Haven Department of Police Services and the Yale University Child Study Center, brings together police officers and mental health professionals for joint training, consultation, and support. This partnership enables them to provide direct, interdisciplinary intervention to children and families who are victims, witnesses, or perpetrators of violent crimes. Law enforcement can refer cases to the Child and Family Traumatic Stress Intervention (CFTSI), designed for the peritraumatic period.

**Results:**

CFTSI focuses on enhancing the caregiver’s understanding and response to both their own and their child’s traumatic reactions. It also aims to improve the child’s comprehension of their emotional responses. The intervention includes establishing strategies to address trauma responses and assessing pre-existing vulnerabilities to determine the need for long-term treatment. Studies have shown that CFTSI is effective in reducing post-traumatic stress symptoms in children and adolescents. A randomized controlled trial (RCT) published in *Child Abuse & Neglect* found that children who received CFTSI had significantly fewer post-traumatic stress symptoms compared to those who received standard care.

**Conclusions:**

Early intervention like this can make a significant difference in the lives of affected children, helping to mitigate the long-term impact of trauma and promote healing and resilience.

**Disclosure of Interest:**

None Declared

